# Modulation of Rumen Microbes Through Extracellular Vesicle Released by the Rumen Fluke *Calicophoron daubneyi*

**DOI:** 10.3389/fcimb.2021.661830

**Published:** 2021-04-20

**Authors:** Nathan R. Allen, Aspen R. Taylor-Mew, Toby J. Wilkinson, Sharon Huws, Helen Phillips, Russell M. Morphew, Peter M. Brophy

**Affiliations:** ^1^ Institute of Biological, Environmental and Rural Sciences, Aberystwyth University, Aberystwyth, United Kingdom; ^2^ Institute for Global Food Security, School of Biological Sciences, Queen’s University Belfast, Belfast, United Kingdom

**Keywords:** *Calicophoron daubneyi*, extracellular vesicle, proteomics, rumen microbiome, mass spectrometry

## Abstract

Parasite derived extracellular vesicles (EVs) have been proposed to play key roles in the establishment and maintenance of infection. *Calicophoron daubneyi* is a newly emerging parasite of livestock with many aspects of its underpinning biology yet to be resolved. This research is the first in-depth investigation of EVs released by adult *C. daubneyi.* EVs were successfully isolated using both differential centrifugation and size exclusion chromatography (SEC), and morphologically characterized though transmission electron microscopy (TEM). EV protein components were characterized using a GeLC approach allowing the elucidation of comprehensive proteomic profiles for both their soluble protein cargo and surface membrane bound proteins yielding a total of 378 soluble proteins identified. Notably, EVs contained Sigma-class GST and cathepsin L and B proteases, which have previously been described in immune modulation and successful establishment of parasitic flatworm infections. SEC purified *C. daubneyi* EVs were observed to modulate rumen bacterial populations by likely increasing microbial species diversity *via* antimicrobial activity. This data indicates EVs released from adult *C. daubneyi* have a role in establishment within the rumen through the regulation of microbial populations offering new routes to control rumen fluke infection and to develop molecular strategies to improve rumen efficiency.

## Introduction

Paramphistomes, commonly known as rumen fluke, have been found to infect ruminant animals worldwide (Huson et al., 2017; Huson et al., 2018). Within tropical and sub-tropical regions, rumen fluke infections cause significant production losses; yet only in recent years have rumen fluke infections been observed throughout Europe with the major species responsible confirmed as *Calicophoron daubneyi* (Sanabria and Romero, 2008; Jones et al., 2017). Clinical disease *via* adult rumen fluke is rarely reported in temperate areas, but mortality to large burdens of immature parasites has been observed in adolescent sheep and cattle (Mason et al., 2012; Millar et al., 2012).

Parasitic helminths establish long-term infections by manipulating the immune system in order to create an anti-inflammatory environment within the host (Coakley et al., 2016; Maizels and McSorley, 2016). In recent years parasite extracellular vesicles (EVs) have been recognized as key components of this strategy by transporting immunomodulatory cargo molecules (Marcilla et al., 2012). To date, there is fragmented understanding of the mechanisms underpinning EV activity with respect to immune-modulation and successful establishment of infection (Coakley et al., 2016). EVs appear the major route for macromolecule exportation from parasitic helminths, with some EVs even containing host mimicking components (Marcilla et al., 2012). EVs released by several helminth parasites have been found to deliver bioactive molecules and miRNA to host cells where they modulate host gene expression and suppress cytokine formation (Buck et al., 2014). The packaged cargo is developmentally regulated likely allowing parasite migration and establishment within the definitive host (Marcilla et al., 2012; Montaner et al., 2014; Cwiklinski et al., 2015). EVs from parasitic flatworms contain a number of established immune modulating proteins, such as FhGST-S1, the Sigma class GST (Prostaglandin synthase) from *F. hepatica* (LaCourse et al., 2012; Davis et al., 2019).

EVs have been confirmed to be released from the rumen fluke *C. daubneyi* (Huson et al., 2018). However, these EVs are yet to be studied at a molecular level or in relation to their effects on rumenal microbes. Previous studies of helminth parasites have shown they interact with their hosts gut microbiota in order to successfully establish infection whilst interrupting the ‘healthy’ microbiome that ultimately promotes the hosts health (Jenkins et al., 2018). With this in mind it is notable that relationships between gut microbiota and parasites are not fully resolved. However, evidence suggests the microbiotas involvement in regulation of the immune system ensuring appropriate responses to pathogenic organisms. However, evidence suggests the microbiotas involvement in regulation of the immune system ensuring appropriate responses to pathogenic organisms (Gensollen et al., 2016). Currently, studies into domestic livestock’s microbiota in response to helminth infections remain limited and inconsistent (Peachey et al., 2019). Specifically, the rumen microbiota has been extensively studied due to the importance of rumen microbes in the nutrition and health of the animal (Petri et al., 2013; Chaucheyras-Durand and Ossa, 2014). Growing evidence suggest the rumen microbiome is involved in a complex and intimate dialogue with the immune and metabolic functions of the host (Zaiss and Harris, 2016). Owing to the links between rumen microbiota and animal health, disturbances in the rumen ecosystem may hinder rumen functionality and lead to disease in the host (Zaiss and Harris, 2016), with studies showing a causal link between natural and experimental infections of parasitic helminths with qualitative and quantitative alterations to the intestinal microbiota in a variety of animal species (Walk et al., 2010; Broadhurst et al., 2012; Li et al., 2012; Cantacessi et al., 2014; Lee et al., 2014). Here we unravel the proteomic profile of adult *C. daubneyi* EVs and explore the EV impact on the complex microbial environment contributing to their successful establishment within the host.

## Methods

### *Calicophoron daubneyi* Collection and *In Vitro* Maintenance

Adult *C. daubneyi* were retrieved from naturally infected bovine rumens post-slaughter in a local abattoir (mid-Wales, UK). Following collection, *C. daubneyi* were washed in phosphate buffered saline (PBS), pH 7.4, at 39°C to remove contaminating materials. Flukes were divided into batches of 30 adults and placed in 1 ml/fluke DME culture media (supplemented with 2.2 mM Ca, 2.7 mM MgSO_4_, 61.1 mM glucose, 1 µM serotonin and gentamycin (5 µg/ml), 15 mM HEPES), 39°C for 6 hours. Subsequently, both flukes and DME media were snap frozen in liquid nitrogen and stored at -80°C.

### EV Purification

Prior to differential centrifugation and size exclusion chromatography EV purification, media was submitted to centrifugation at 300 *× g* for 10 minutes at 4°C, followed by centrifugation at 700 *× g* for 30 minutes at 4°C to remove residual debris.

### Differential Centrifugation (DC)

*C. daubneyi* maintenance media was utilized in order to purify EV populations through differential centrifugation (DC) as previously described (Davis et al., 2019). Media was centrifuged at 120,000 *× g* for 80 min at 4°C in an Optima L-100 XP ultracentrifuge (Beckmann Coulter, High Wycombe, UK). The resulting pellet was washed in 5 ml PBS, pH 7.4 and submitted to 0.2 µm syringe filtering before the centrifugation step being repeated. The resulting pellet was suspended in 500 µl PBS and stored at -20°C.

### Size Exclusion Chromatography (SEC)

*C. daubneyi* maintenance media was utilized in order to purify EV populations through size exclusion chromatography (SEC) as previously described (Davis et al., 2019). Media was concentrated using Amicon ultra-15 centrifugal filters (Merk, Millipore), with a 10 kDa MW cut off. Samples were added to the centrifugal unit and centrifuged at 4000 *× g* for 20 min at 4°C until an approximately 500 µl EV enriched sample remained. EV enriched samples were 0.2 µm filtered and a maximum of 500 µl passed through qEVoriginal SEC columns (IZON science, U.K) following the manufacturers protocol. Briefly, columns were equilibrated with a minimum of 10 ml of PBS prior to addition of the sample. The initial 2.5 ml flow through was discarded with the following 2.5 ml EV enriched fraction retained and stored at -20°C.

### Quantification Using Tunable Resistive Pulse Sensing

A Nanopore NP200 (IZON Science) was utilized in the quantification of SEC purified EV samples. The Nanopore was calibrated using calibration particles (CPC200, 1:1000 filtered PBS). EV samples were measured at 47 mm nanopore stretch at a 100 nA voltage under 7 mbar pressure. Particles were detected through short pulses of current and the resulting data analyzed using qNano particle analysis software (IZON, version 3.2).

### Transmission Electron Microscopy (TEM)

DC and SEC purified EVs were fixed onto formvar/carbon coated copper grids (Agar Scientific) for TEM analysis following the manufacturer’s instructions. Briefly, 10 µl of EV-enriched sample was added to each grid and incubated for 45 min on ice. Grids were placed on the meniscus of 4% w/v uranyl acetate for 5 min on ice. Grids were then stored for a minimum of 24 hours at room temperature prior to visualization on the TEM (Jeol JEM1010 microscope at 80 kV), with EV presence confirmed through size selective criteria (30 – 200nm).

### EV Proteomic Analysis

EV proteins were determined through sodium dodecyl sulfate polyacrylamide gel electrophoresis (SDS-PAGE) following the method of Laemmli (1970). Protein concentration was first determined using a Qubit protein assay following the manufacturer’s instructions (Thermo Scientific, UK). Loading concentrations of 10 µg were aliquoted and centrifuged at 100,000 *× g* at 4°C for 30 minutes (S55-S rotor, Sorval MX120 centrifuge, Thermo scientific) with the resulting supernatant discarded. The EV pellet was suspended in 10 µl loading buffer and heated for 10 min at 95°C before loading into hand-cast 7 cm x 7 cm 12.5% polyacrylamide Tris/glyceine gels and subject to electrophoresis on a Protean III system (Bio-Rad, UK). Tris/Glycine/SDS buffer (25 mM Tris, 192 mM Glycine, 0.1% w/v SDS pH 8.3) (BioRad, U.K) was utilized for electrophoresis, with gels run at 70 V through the stacking gel and 150 V until completion. Gels were fixed (40% v/v ethanol and 10% v/v acetic acid) for one hour prior to overnight staining with colloidal Coomassie Brilliant Blue (Sigma, UK) at room temperature with gentle agitation. De-staining was achieved using 30% (v/v) methanol, 10% v/v acetic acid and gels were subsequently visualized on a GS-800 calibrated densitometer (Bio-Rad, UK) and stored in 1% acetic acid prior to trypsin digestion for mass spectrometry analysis following the protocol of Davis et al. (2019).

### EV Surface Trypsin Hydrolysis

SEC purified EVs were concentrated to a final concentration of 200 µg in 250 µl. Sequencing grade trypsin (Roche, U.K) was diluted to 100 µg/ml and added to the EVs resulting in a final concentration of 50 µg/ml. Samples were incubated for 5 minutes at 37°C followed by centrifugation for 1 hour at 100,000 *× g* at 4°C (S55-S rotor, Sorval MX120 centrifuge, Thermo Scientific). The resulting supernatant was divided into 20 µl fractions and subject to LC MSMS with an injection volume of 1 µl.

### Mass Spectrometry

Trypsin digested protein samples were suspended in 20 µl 0.1% formic acid and loaded into an Agilent 6550 iFunnel Q-TOF mass spectrometer combined with a Dual AJS ESI source 1290 series HPLC system (Agilent, Cheshire, U.K). A Zorbax Eclipse Plus C18 column (2.1 x 50 mm 1.8 micron) was utilized with each sample injected into an enrichment column within the system at a flow rate of 2.5 µl/min using an automated micro sampler with an injection volume of 2 µl in the resuspension buffer 0.1% v/v formic acid and allowed to separate at 300 nl/min. Enrichment and separation were carried out on a polaris chip (G4240-62030, Agilent Technologies, U.K). A system of solvents was utilized over the process, solvent A (milliQ water containing 0.1% formic acid) and solvent B (90% v/v acetonitrile containing 0.1% v/v formic acid). Chromatography was achieved using a linear-gradient of 3-8% solvent B over 6 seconds, 8-35% solvent B over 15 minutes, 35-90% solvent B over five minutes and finally 90% solvent B for two minutes. Resulting peak spectra data was loaded onto Agilent Qualitative analysis software (Agilent technologies LDA UK Limited, UK). Each file had compounds found by molecular feature and were saved to MGF. MASCOT (www.matrixofscience.com) was used for analysis by carrying out an MS/MS ion search, settings were set for the enzyme trypsin – allowing 2 missed cleavages, with a fixed modification of carbamidomethyl (C) and a variable modification of oxidation (M) with a peptide charge of 2+, 3+ and 4+. Each sample was then searched against an in-house database composed of a transcript for *C. daubneyi* (Huson et al., 2018) available to search at https://sequenceserver.ibers.aber.ac.uk/. Each of the contigs returned were then searched within an in-house copy of the transcript and the nucleotide sequence recorded. All of the contigs were then translated using ExPasy (www.expasy.com) and the sequences submitted to BLASTp analysis and subsequently searched in the Interpro database.

### EV Rumen Microbe Interactions

Rumen contents were collected from rumen-fistulated steers at Trawsgoed experimental farm (Aberystwyth, Wales) complying with the authorities of the UK Animal (Scientific Procedures) Act (1986). Rumen contents was squeezed through a sieve allowing retention of strained ruminal fluid (SRF) that was immediately incubated at 39°C. Rumen fluid was added to anaerobic incubation medium following the protocol of Goering and Van Soest (1970), to create a 10% v/v solution. PBS was removed from SEC purified EV samples (5.52E+10 particles/ml) through centrifugation using Amicon ultra-15 centrifugal filters (Merk, Millipore) 10 kDa MWCO, with EVs resuspended in an equal volume of modified Van Soest digestion buffer. 1 ml EV solution was added to 9 ml rumen fluid/anaerobic incubation medium (n =3) and allowed to incubate for 24 hrs. For controls, EV solution was replaced with modified Van Soest digestion buffer. Rumen fluid sampling was carried out at 5 time points during the incubation period (0 h, 2 h, 4 h, 6 h, and 24 h) and stored for downstream qPCR analysis.

### Rumen Fluid DNA Extraction

DNA extractions were carried out on 1 ml rumen fluid from each of the aforementioned time points (0 hrs, 2 hrs, 4 hrs, 6 hrs and 24 hrs). Extractions were carried out using a FastDNA spin kit for soil (MP Biomedicals, USA) according to the manufacturers protocol, as described by Huws et al. (2010). Extracted DNA was quantified using the Biotech Epoch Microplate Spectrophotometer (Biotek Instruments Inc, USA). The Epoch Microplate Spectrophotometer was calibrated prior to quantification using 1.25 µl DNase/pyrogen free water. Following quantification samples were stored at -20°C for qPCR analysis.

### Bacterial qPCR Analysis

qPCR of 16S rDNA was undertaken to determine the effects of incubation of EVs with rumen fluid on total bacteria population as well as specifically *Ruminococcus albus, Fibrobacter succinogenes*, and *Prevotella* spp. Extracted DNA was diluted 10-fold with ddH_2_O. The reaction mixture (1215 µl) for each qPCR run was prepared with 1 x SYBR Geen I master mix (Applied Biosystems), 5.4 µl of each primer (**Table 1**) and ddH_2_0. 10 µl of reaction mixture was added to 1 µl of each DNA sample analyzed using a Roche lightcycler 480 II (Roche diagnostics Ltd.) on a 384 well qPCR plate. A bacterial standard was prepared with equal amounts of genomic DNA as outlined by Huws et al. (2010). For each qPCR, with the exception of *Prevotella* spp., amplification was performed at 95°C for 10 minutes, followed by 35 cycles of 95°C for 15 seconds, 58°C for 15 seconds, and 72°C for 15 seconds, and then an extension step of 72°C for 5 minutes. For the *Prevotella* spp. qPCR amplification was performed at 95°C for 10 minutes followed by 35 cycles of 95°C for 15 seconds, 55°C for 15 seconds, and 72°C for 15 seconds, and then an extension step of 72°C for five minutes. All qPCR reactions were performed in triplicate and assay qPCR efficiency was calculated as: efficiency=10(- 1/slope) x100. The bacterial standards were used to create a standard curve to allow for quantification of the samples. Statistical analysis of the qPCR values was undertaken in Microsoft Excel, and using repeated measures ANOVA to test for significant differences in IBM SPSS Statistics 23.0.

**Table 1:** Forward and reverse primer sequences used to target 16S rDNA in qPCR analysis of total bacteria, *Ruminococcus albus, Fibrobacter succinogenes* and *Prevotella* spp. DNA concentrations.

Target	Forward primers (5’-3’)	Reverse primers (3’-5’)
**Total Bacteria**	GTGSTGCAYGGYTGTCGTCA	GAGGAAGGTGKGGAYGACGT
***Ruminococcus albus***	CCCTAAAAGCAGTCTTAGTTCG	CCTCCTTGCGGTTAGAACA
***Fibrobacter succinogenes***	GGTATGGGATGAGCTTGC	GCCTGCCCCTGAACTATC
***Prevotella* spp.**	CACRGTAAACGATGGATGCC	GGT CGG GTT GCA GAC C

## Results

### Confirmation of EVs in Adult *C. daubneyi* Maintenance Media

**Figure 1:**
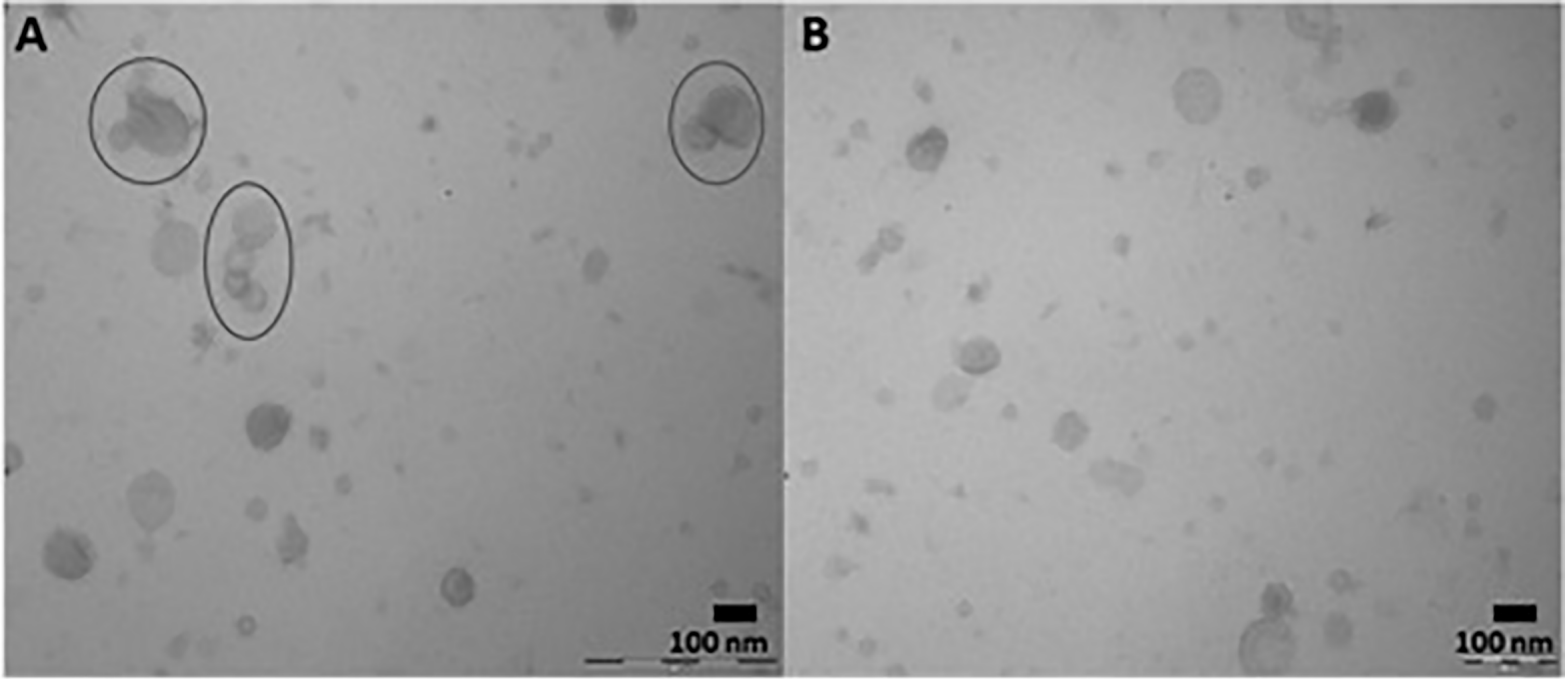
Representative developed TEM micrographs identifying extracellular vesicles secreted by *C. daubneyi in vitro* through DC and SEC isolation. **(A)** DC purified samples with visible aggregation of vesicles (circled) **(B)** SEC purified samples demonstrating a reduction in EV aggregation.

The presence of extracellular vesicles in both DC and SEC purified adult *C. daubneyi* samples was confirmed by the identification of membrane bound vesicles ~30-100 nm in size through transmission electron microscopy (TEM). TEM imaging demonstrated EVs present to have diverse morphologies with ruptured vesicles only identified in DC purified samples. A large number of aggregated vesicles were also observed in DC samples (**Figure 1A**) whilst reduced aggregation was observed in the samples isolated through SEC (**Figure 1B**) and, despite the inclusion of 0.2 µm filtering, background contamination was visibly present following both purification methods.

### Adult *C. daubneyi* Whole EV Proteome

**Figure 2:**
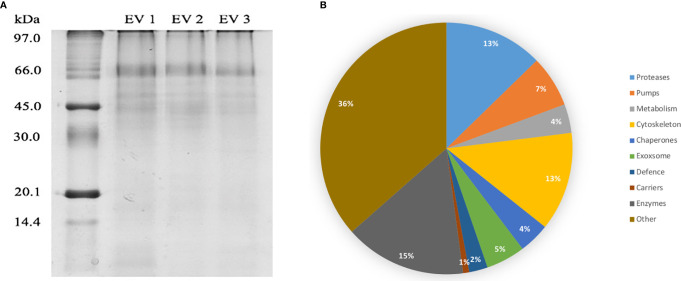
**(A)** EV proteome arrays of lysed *C. daubneyi* EVs (n=3). EVs released *in vitro* were lysed and subjected to 12.5% 1D polyacrylamide gel analysis and colloidal Coomassie blue stained. All biological replicates produced a highly reproducible profile. **(B)** Categorization of all sequences returned from the *C. daubneyi* EV proteome. Proteins consistent across three replicates were submitted to Interpro and GeneOntology searches and assigned to 9 functional categories as defined by Cwiklinski et al. (2015). Proteins that did not fit any of the nine categories were placed into a final category classified as ‘other’. Cytoskeleton associated proteins accounted for 13% of the sequences resolved, Proteases 14%, Enzymes 8%, Chaperones 7%, Transporters 4%, Exosome biogenesis 3%, Metabolism 5%, Carriers 1% with Others filling the remaining 36%.

DC purified EVs were utilized to resolve the *C. daubneyi* whole lysed EV proteome. A GelC strategy was exploited to identify lysed EV proteins with proteins resolved on a 12.5% one-dimensional sodium dodecyl sulfate-polyacrylamide gel (SDS-PAGE) followed by LC-MSMS analysis with the mass spectrometry proteomics data deposited to the ProteomeXchange Consortium *via* the PRIDE partner repository with the dataset identifier PXD024182. Replication of lysed EV proteomic profiles confirmed reproducibility (**Figure 2A**). A total of 378 proteins were found to be consistent across three biological replicates (n = 3) following LC-MSMS analysis (**Supplementary Material 1**). EV protein abundance was quantified by the number of unique peptides present, with only protein hits above the significance threshold (>47) included as a positive identification. Quantification by the number of unique peptides elucidated the top 50 protein hits (**Table 2**). Further analysis of the returned proteome identified a number of common EV markers through comparison with the Exocarta database (http://exocarta.org). All 378 sequences resolved in the EV proteome were further characterized by their functionality through use of the Interpro database and sorted into 9 distinct categories: Cytoskeleton, Proteases, Enzymes, Chaperones, Metabolism, Transporters, Carrier, Exosome Biogenesis and Others as previously described by (Cwiklinski et al., 2015) (**Figure 2B**). Interestingly, the category with the greatest number of sequences assigned was ‘other’ encompassing all sequences with no BLAST result or a BLAST result to a hypothetical or unassigned protein accounting for 36% of the sequences. This was followed by cytoskeletal proteins accounting for 24% of proteins. The category representing the fewest number of proteins were carriers accounting for 1% of the total proteome.

**Table 2:** Top 50 proteins resolved in *C. daubneyi* extracellular vesicles following BLAST analysis of transcript identifiers.

Transcript ID	Isoform	Unique peptides	Blast description	Organism	NCBI accession
TR26097|c0_g1	i1	76	ATPase family protein	*Opisthorchis viverrini*	OON14744.1
TR18968|c0_g1	i1	58	Tubulin beta-3	*Fasciola hepatica*	CAP72051.1
TR17099|c2_g1	i1	57	Tubulin beta	*Clonorchis sinensis*	GAA51682.1
TR21569|c0_g5	i1	44	No hit	No hit	No hit
TR9358|c0_g1	i1	39	Actin	*Gossypium arboreum*	XP_017626052.1
TR19715|c0_g1	i1	36	Radixin	*Carlito syrichta*	XP_008054748.1
TR21569|c0_g5	i2	36	No hit	No hit	No hit
TR17877|c2_g2	i1	31	Alpha-tubulin	*Fasciola hepatica*	CAO79602.1
TR18958|c0_g1	i1	28	Alpha tubulin	*Schistosoma japonicum*	AAW27478.1
TR19159|c0_g1	i1	26	Alpha tubulin	*Clonorchis sinensis*	GAA56421.1
TR23254|c0_g1	i1	24	Leucyl aminopeptidase	*Clonorchis sinensis*	ABL11479.1
TR24554|c0_g1	i1	23	alpha-glucosidase	*Schistosoma mansoni*	XP_018647945.1
TR18070|c0_g1	i1	22	Acid sphingomyelinase phosphodiesterase	*Clonorchis sinensis*	GAA33847.2
TR23757|c0_g1	i1	22	Alpha tubulin	*Clonorchis sinensis*	GAA38337.2
TR24153|c0_g1	i1	22	Hypothetical protein	*Opisthorchis viverrini*	OON14506.1
TR23969|c0_g1	i1	21	Tektin	*Clonorchis sinensis*	GAA33438.1
TR23279|c0_g1	i1	21	Alpha tubulin	*Fasciola hepatica*	CAO79606.1
TR18525|c0_g1	i1	20	14-3-3 epsilon	*Opisthorchis viverrini*	OON22058.1
TR21014|c0_g1	i1	20	SNaK1	*Schistosoma mansoni*	AAL09322.1
TR20466|c0_g1	i1	19	Calpain	*Schistosoma mansoni*	CCD74981.1
TR20643|c0_g1	i1	18	annexin a7	*Schistosoma haematobium*	KGB33756.1
TR18939|c0_g1	i1	18	No hit	No hit	No hit
TR22034|c1_g4	i3	18	Aldolase	*Opisthorchis viverrini*	OON20700.1
TR25036|c3_g1	i13	17	Cathepsin D	*Fasciola gigantica*	AEE69372.1
TR25036|c3_g1	i2	17	Cathepsin D	*Fasciola gigantica*	AEE69372.1
TR23288|c0_g1	i1	16	EF-hand domain	*Schistosoma mansoni*	CCD76447.1
TR19073|c1_g2	i1	16	Hypothetical protein	*Opisthorchis viverrini*	OON16570.1
TR18454|c0_g1	i1	16	Hypothetical protein	*Opisthorchis viverrini*	OON20759.1
TR25036|c3_g1	i1	16	eukaryotic aspartyl protease	*Opisthorchis viverrini*	OON23093.1
TR21569|c0_g5	i7	16	No hit	No hit	No hit
TR25036|c3_g1	i14	14	Cathepsin D	*Clonorchis sinensis*	GAA56870.1
TR23782|c0_g2	i1	14	Leukotriene-A4 hydrolase	*Clonorchis sinensis*	GAA49617.1
TR17046|c0_g1	i1	15	14-3-3 epsilon	*Clonorchis sinensis*	AEO89649.1
TR9216|c0_g1	i1	15	14-3-3 protein	*Opisthorchis viverrini*	OON14987.1
TR25395|c0_g2	i2	15	Hypothetical protein	*Opisthorchis viverrini*	XP_009165006.1
TR17779|c0_g1	i1	15	Actin	*Opisthorchis viverrini*	XP_009173847.1
TR25395|c0_g1	i1	15	Hypothetical protein	*Opisthorchis viverrini*	XP_009165006.1
TR22003|c1_g5	i1	14	Tubulin beta	*Cricetulus griseus*	XP_007606483.1
TR19892|c0_g1	i1	14	Hypothetical protein	*Opisthorchis viverrini*	OON16605.1
TR18374|c0_g1	i1	14	Triose phosphate isomerase	*Fasciola hepatica*	AGJ83762.1
TR25036|c3_g1	i12	14	Cathepsin E-A	*Apaloderma vittatum*	KFP91951.1
TR17173|c0_g1	i1	14	leishmanolysin peptidase	*Clonorchis sinensis*	GAA54636.1
TR17164|c0_g1	i1	13	glyceraldehyde 3- phosphate dehydrogenase	*Clonorchis sinensis*	GAA28380.1
TR15297|c0_g1	i1	13	Chloride intracellular channel	*Clonorchis sinensis*	GAA38512.2
TR19675|c0_g1	i1	13	JF-2	*Schistosoma japonicum*	AAB49033.1
TR25036|c3_g1	i8	13	Cathepsin D	*Clonorchis sinensis*	GAA56870.1
TR23072|c0_g1	i1	13	EF-hand calcium-binding domain	*Clonorchis sinensis*	GAA51832.1
TR24199|c0_g4	i1	12	Plastin-1	*Clonorchis sinensis*	GAA29911.1
TR21252|c0_g1	i1	12	33kDa inner dynein arm light chain	*Schistosoma japonicum*	CAX73643.1
TR25837|c0_g3	i3	12	EF-hand domain-containing family member	*Clonorchis sinensis*	GAA35263.2

Proteins were ranked based on the number of unique peptides sequenced during tandem LC-MSMS and BLAST identifiers chosen based on E-values.

### Trypsin Hydrolysis of External Surface Proteins on Adult *C. daubneyi* EVs

Following resolution of the whole EV proteomic profile, the proteins present on the external surface of EVs were investigated through trypsin cleavage from the membrane. Transcript IDs identified through LC-MSMS were translated before submission to BLASTp investigation allowing identification of protein IDs. In total, 89 proteins were identified as present upon the external surface of EVs release by adult *C. daubneyi* (**Table 3**), including a variety of well-known exosomal markers such as heat shock protein 70 and members of the tetraspanin family as defined by the Exocarta database (http://www.exocarta.com). Several membrane channel and transporter proteins were identified including ATPase, V-type H+- transporting ATPase, phospholipase and glucose transporters.

**Table 3:** Putative proteins identified in SEC purified *C. daubneyi* EVs surface trypsin shave (n = 3).

Transcript ID	Blast ID
TR19715|c0_g1_i1	Moesin ezrin radixin homolog 1 isoform X1
TR18070|c0_g1_i1	Acid sphingomyelinase-like phosphodiesterase 3a
TR20146|c0_g1_i2	No hit
TR9358|c0_g1_i1	Actin-7
TR17099|c2_g1_i1	Tubulin beta chain
TR18542|c0_g1_i1	Tubulin beta chain
TR22003|c1_g6_i1	Tubulin beta-2C chain
TR22003|c1_g4_i3	Tubulin beta chain isoform X1
TR23322|c0_g3_i1	Tubulin beta-2C chain
TR22003|c1_g2_i1	Beta tubulin
TR22003|c0_g1_i1	Tubulin beta chain
TR21569|c0_g5_i1	No hit
TR21569|c0_g5_i2	No hit
TR25036|c3_g1_i12	Cathepsin E-
TR25036|c3_g1_i14	Lysosomal aspartic protease
TR3846|c0_g1_i1	Cathepsin D (lysosomal aspartyl protease)
TR6048|c0_g1_i1	Asparticase oryzasin-1-like
TR25036|c3_g1_i1	Cathepsin E-A-like
TR25036|c5_g1_i1	Renin
TR25036|c0_g1_i1	Lysosomal aspartic protease-like
TR25036|c3_g1_i2	Cathepsin D (lysosomal aspartyl protease)
TR25036|c3_g1_i8	Lysosomal aspartic protease-like
TR55450|c0_g1_i1	No hit
TR16856|c0_g1_i1	DM9 domain-containing
TR18939|c0_g1_i1	No hit
TR17640|c0_g1_i1	No hit
TR20530|c0_g1_i1	Heat shock 90
TR21065|c0_g1_i1	Heat shock 75 mitochondrial
TR24356|c0_g1_i3	Program cell death 6-interacting
TR15792|c0_g1_i1	Golgi-associated plant pathogenesis-related 1
TR23254|c0_g1_i1	Leucyl aminopeptidase
TR17173|c0_g1_i1	Leishmanolysin-like peptidase
TR19239|c0_g1_i1	No hit
TR12225|c0_g1_i1	Erythrocyte band 7 integral membrane
TR16040|c0_g1_i1	Lysosomal Pro-X carboxypeptidase precursor
TR18162|c0_g1_i1	Liver basic fatty acid binding
TR26002|c2_g1_i1	Cytoplasmin type 5
TR33621|c0_g1_i1	No hit
TR29071|c0_g1_i1	Actin
TR4440|c0_g1_i1	No hit
TR23598|c0_g2_i1	Adenylate kinase 9
TR17877|c2_g2_i1	Tubulin alpha-1A chain-like
TR19159|c0_g1_i1	Tubulin alpha-1A chain
TR18958|c0_g1_i1	Tubulin alpha-1A chain-like
TR12612|c0_g1_i1	Alpha tubulin
TR21082|c0_g1_i1	Tubulin GTPase domain
TR17328|c0_g1_i1	Na(+) H(+) exchange regulatory cofactor NHE-RF1
TR20466|c0_g1_i1	Leucine-rich repeat-containing 23
TR9216|c0_g1_i1	Tyrosine 3-monooxygenase tryptophan 5-monooxygenase
TR16536|c0_g1_i1	14-3-3 beta alpha-1
TR17046|c0_g1_i1	14-3-3 epsilon
TR20794|c0_g1_i1	Phosphoglycerate kinase 1
TR23598|c0_g1_i1	Adenylate kinase 9-like
TR20586|c0_g1_i2	Regulator of microtubular dynamics 1-like
TR13665|c0_g1_i1	Calcyphosin isoform X5
TR19675|c0_g1_i1	Radixin isoform X1
TR20928|c0_g1_i1	Cathepsin B-like cysteine ase precursor
TR11284|c0_g1_i1	Histone H4
TR16097|c0_g1_i1	Chloride intracellular channel 4
TR16168|c0_g1_i1	Actin depolymerizing factor
TR15827|c0_g1_i1	Lysosomal protective
TR17138|c0_g1_i1	Fatty acid binding brain
TR17367|c0_g1_i1	Enolase
TR19538|c0_g1_i1	Charged multivesicular body 1a
TR22854|c0_g1_i4	Aquaporin-1
TR15761|c0_g1_i1	Lysosomal alpha-glucosidase
TR20893|c0_g1_i1	Methylthioadenosine phosphorylase
TR12782|c0_g1_i1	8 kDa calcium-binding
TR36972|c0_g1_i1	Histone H4-like
TR18466|c1_g2_i1	Globin-3
TR20091|c0_g1_i1	Glucose transport
TR17869|c0_g1_i1	Phospholipase D3
TR15896|c0_g1_i1	Calmodium 6
TR17164|c0_g1_i1	Glyceraldehyde 3-phosphate dehydrogenase
TR17741|c0_g1_i1	Heat shock 70
TR22803|c1_g1_i2	Annexin A11
TR3136|c0_g1_i1	No hit
TR20643|c0_g1_i1	Annexin A7
TR17762|c0_g1_i2	Lysosomal acid phosphatase
TR16407|c0_g1_i2	Cathepsin D (lysosomal aspartyl protease)
TR22152|c0_g1_i1	Hypothetical protein CLF_104825
TR16514|c0_g1_i1	No hit
TR23279|c0_g1_i1	Tubulin alpha testis-specific
TR18133|c0_g1_i3	CD63 antigen

Including transcript identifiers and BLAST description. The top BLAST hit was chosen based on the lowest E-value and transcripts were ordered by number of unique peptides.

### EV Interaction With the Rumen Microbiome

**Figure 3:**
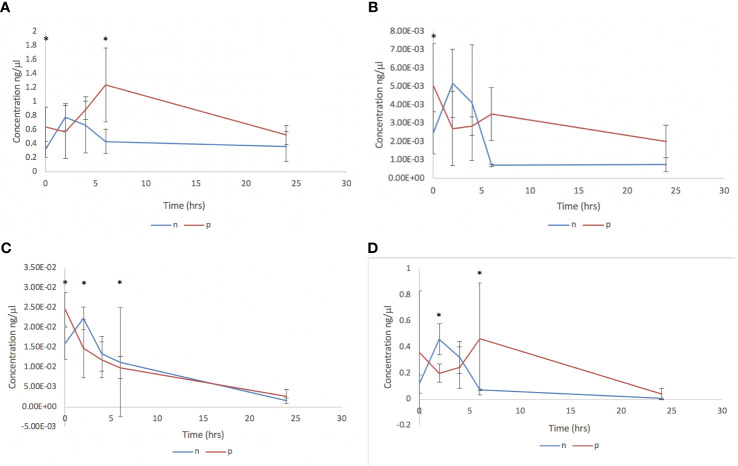
Bacterial qPCR analysis following *in vitro* culture of rumen fluid incubated with *C. daubneyi* SEC purified EVs (p) and without EVs (n). Data shown for **(A)** Total bacteria, **(B)** *Fibrobacter succinogenes*, **(C)** *Ruminococcus albus* and **(D)** *Prevotella* spp. *indicates a significant difference between treatment means.

The impact of adult *C. daubneyi* EVs on rumen microbial populations was completed on three key ruminant bacterial species, *Fibrobacter succinogenes, Ruminococcus albus* and *Prevotella spp*, as well as on the total bacterial microbiome using 16S rDNA analysis. Quantitative PCR was undertaken on samples at five timepoints (0 hrs, 2 hrs, 4 hrs, 6 hrs and 24 hrs) to assess the impact of EVs on the rumen microbiome (**Figure 3**). Total Bacteria DNA concentrations ranged from 500.93 to 1236.95 ng/ml in cultures incubated in the presence of rumen fluke EVs, and from 229.93 to 656.22 ng/ml in cultures with absence of rumen fluke EVs. *Fibrobacter succinogenes* DNA concentration ranged from 0.471 to 7.871 ng/ml with EVs and from 0.207 to 5.384 ng/ml in the absence of EVs. *Ruminococcus albus* DNA concentrations ranged from 0.175 to 0.509 ng/ml with EVs, and from 0.049 to 0.526 ng/ml in the absence of rumen fluke EVs. Finally, the DNA concentrations for *Prevotella* spp. ranged from 70.60 to 298.03 ng/ml with EVs, and from 30.45 to 326.97 ng/ml in the absence of rumen fluke EVs.

In terms of overall treatment, EV presence or absence, a significant effect of treatment with *C. daubneyi* EVs was only observed for total bacteria (P = 0.002). Whereby, the incubation of rumen fluid with EVs led to an increase in total bacterial concentration, with no significant overall effect of treatment on bacterial concentrations for *F. succinogenes*, *R. albus*, or *Prevotella* spp. Analysis of overall treatment between time points (0-24 hrs) showed significant interaction for total bacteria (P=0.008), *R. albus* (P<0.05), and *Prevotella* spp. (P<0.05). However, there was no significant interaction between timepoint and treatment for *F. succinogenes* (P>0.05). For total bacteria concentrations, there was a significant difference between treatment means at the zero hours and six-hour timepoints. For *F. succinogenes*, the only timepoint at which there was a significant difference between treatment means was at zero hours (P=0.001). There was a significant difference in the mean concentration of *R. albus* DNA between treatments at all except the four-hour timepoint. *Prevotella* spp. had a significant difference between treatment means at the two and six hour timepoints (P >0.01).

## Discussion

Utilization of the recently reported adult *C. daubneyi* transcriptome (Huson et al., 2018) has allowed a comprehensive proteomic characterization of the adult helminth’s membrane bound vesicle secretions, leading to identification of 378 proteins consistent across biological replicates. Comparison with resolved eukaryote EV proteomes highlighted a number of common proteins including, tetraspanins (TR20913|c0_g1_i1, TR22166|c0_g1_i1, TR22094|c0_g1_i1 and TR22869|c0_g1_i12), Heat shock proteins (TR17741|c0_g1_i1 and TR20530|c0_g1_i1) and Annexins (TR22803|c1_g1_i2, TR20643|c0_g1_i1 and TR17648|c0_g1_i1). This is in addition to EV associated cytoskeletal proteins such as Actin (TR9358|c0_g1_i1, TR17779|c0_g1_i1 and TR28482|c0_g1_i1) and Ezrin (TR19715|c0_g1_i1) as well as proteins involved in metabolic processes such as enolase (TR17367|c0_g1_i1, TR24268|c0_g1_i1, TR24268|c0_g2_i1 and TR19628|c0_g2_i1), Peroxidases (TR17193|c0_g1_i1 and TR12513|c0_g1_i1) and pyruvate kinases (TR21788|c0_g1_i1) (Choi et al., 2013; Nowacki et al., 2015). The consistency in proteins with established EV proteomes further supports the identification of the membrane bound vesicles by TEM imaging as EVs, suggesting the *C. daubneyi* secretome is more complex than previously demonstrated (Huson et al., 2018).

Protein cargo packaged into EVs prior to their release is dependent upon cellular source and release cell associated activity (Simons and Raposo, 2009). Similar to the closely related trematode *F. hepatica* and in contrast to several trematode species such as *E. caproni*, *S. mansoni* and *D. dendriticum*, rumen fluke EVs returned a large quantity of proteases and peptidases including Xaa-pro peptidase, cathepsins and metalloproteases (Marcilla et al., 2012; Bernal et al., 2014; Sotillo et al., 2016). Differences observed in protein cargo packaged between species could be due to their residency within the definitive host but could also be attributed to conditions during time of release (Nowacki et al., 2015). Hits to hypothetical proteins and proteins with ‘no confirmed identity’ highlighted the variability in proteins packaged and their likely roles in parasite establishment that are likely unique to the rumen fluke. In total, 14.2% of the proteins identified represented these undefined proteins and their further investigation could allow insight into infection, migration and successful establishment of infection (Dalton et al., 2003). Consistent to studies in *F. hepatica* a plethora of molecules including fatty-acid binding proteins, sigma-class glutathione transferases and cathepsin B were identified which are known to be internalized by host cells with their immunomodulation activity leading to a TH2-mediated environment that is favorable for parasite establishment (Dalton et al., 2003; Donnelly et al., 2010; Dowling et al., 2010). As with previous trematode studies, the presence of uncharacterized proteins allows the hypothesis that they could contain a plethora of novel sequences with potential roles in parasite pathogenesis. Investigation of uncharacterized proteins with no homology to resolved sequences provides an assortment of possible research avenues into future control and intervention of infection (Mulvenna et al., 2010).

Proteins present upon the surface of parasite derived EVs have been found critical to EV function as they interact directly with cells mediating cellular uptake and affecting immune recognition whilst also allowing identification, isolation and classification of EV subpopulations (Buzás et al., 2018). Trypsin hydrolysis of the surface of *C. daubneyi* EVs identified a total of 86 proteins including a variety of well-known exosomal markers such as heat shock protein 70 and members of the tetraspanin family as defined by the Exocarta database (http://www.exocarta.com). As expected, a trematode specific tetraspanin (CD63) was identified within the proteome, as common EV markers, members of the tetraspanin family have been widely investigated with studies on *O. viverrini* highlighting the potential use of EV derived tetraspanins as vaccine candidates due to their ability to prevent EV uptake and internalization into host cells (Chaiyadet et al., 2015). As with studies into closely related trematode *F. hepatica* EVs, many of the surface proteins resolved represented metabolic enzymes such as enolase, Glyceraldehyde-3-dehydrogenase and annexins with primary roles as adhesion molecules interacting directly with the surface of host cells and so represent possible targets in preventing *C. daubneyi* successful establishment through interruption of EVs internalization by target cells (Bernal et al., 2004; Lama et al., 2009; de la Torre-Escudero et al., 2012).

Following resolution of both the cargo and membrane bound proteome, the potential of *C. daubneyi* EVs to modulate the microbiome were investigated on a range of ruminant bacterial species. Both helminths and bacterial species residing within the gut have been found to have strong immunomodulatory effects on the mammalian host, with a variety of studies showing helminths effect on the microbiota correlating to the helminths successful establishment (Reynolds et al., 2015). Helminths ability to regulate gut microbiota is important due to the ability of certain species to elicit the host immune response favorable for survival (Reynolds et al., 2015), with several previous helminth studies highlighting their ability to regulate bacterial populations within the gut (Su et al., 2018). Here, the effect of EVs on bacterial species encompassed three bacterial species found within the rumen as well as the total bacterial counts within the rumen microbiome. *F. succinogenes*, *R. albus*, and *Prevotella* spp. were chosen for quantification using qPCR to investigate the effect of EVs on the rumen microbiome and any subsequent effects on metabolism. Of these three species, *F. succinogenes* and *R. albus* are considered to be the main cellulolytic bacteria in the rumen (Forsberg et al., 1997), with these species extensively studied using a combination of pure culture and molecular techniques (Minato and Suto, 1978; Mosoni et al., 1997; Koike et al., 2003; Shinkai and Kobayashi, 2007; Zeng et al., 2015). The third species utilized, *Prevotella* spp. represent non-cellulolytic bacteria that play a vital role in ruminal protein degradation (Wallace, 1996; Alauzet et al., 2010).

Exposure of *C. daubneyi* EVs to ruminant bacterial populations showed no significant effect between EV treatment and controls, suggesting there is no significant effect of *C. daubneyi* EVs on rumen microbial facilitated metabolism, or in particular fiber and protein digestion. As expected, given the absence of a feed source, a drop in bacterial DNA concentrations at the 24-hour time point was common across all samples, as feed associated bacteria comprise 70-80% of the ruminal microbial matter (McAllister et al., 1994). The only timepoint found to have a significant difference between treatment means for *F. succinogenes* was timepoint zero. This could be due to the use of rumen fluid inoculum which is deemed as the largest source of variation in *in vitro* rumen studies, due to variations that can occur due to its microbial activity, the preparation method, the concentration of the rumen fluid used, the donor animal from which it is derived and their diet, and even variances within the day have been reported (Cone et al., 1996; Jessop and Herrero, 1998; Rymer et al., 1999; Mould, 2003; Váradyová et al., 2005). For *R. albus* the only timepoint at which there was no significant difference between treatments was the 4-hour timepoint. Whilst, not large enough to be significant the inclusion of EVs appears to slow the decrease in the concentration of *R. albus* over time. A similar effect of EV inclusion was observed for *Prevotella* spp. although again this was not significant.

However, a significant difference was observed for total bacteria DNA concentrations between EV treatment and controls. An increase observed in total bacteria populations alongside no significant differences between treatment and controls for *F. Succinogenes*, *R. albus*, and *Prevotella* spp. may indicate that addition of EVs leads to an increase in total bacterial diversity. This increase in diversity could be due to rumen fluke EVs promoting the survival of several bacterial species in the rumen with previous whole parasite studies reporting increases in certain bacterial species in response to infection. Walk et al. (2010) and Reynolds et al. (2015) observed an increase in members of the lactobacillaceae family in the ileum of mice infected with *H. polygyrus*, despite the mice having different microbiotas present at the outset of the experiment. Similarly, the administration of a single dose of *Trichuris suis* led to a reduction in the abundance of *Fibrobacter* and *Ruminococcus*, accompanied by an increase of campylobacter in gastrointestinal microbiota of pigs (Wu et al., 2012). Alternatively, rumen fluke EVs could be promoting an increase in overall ruminal bacterial diversity as has been observed in gastrointestinal helminth infections in humans (Sepehri et al., 2007; Monira et al., 2012; Lee et al., 2014). Due to the absence of significant differences between treatments and the observed change in total bacteria concentrations the effects of *C. daubneyi* EVs on further ruminal bacterial species would allow a more in-depth understanding of *C. daubneyi* regulation of the microbiota.

Currently, studies into the effects of parasitic helminths on the gut microbiota of their ruminant hosts remain inconsistent, with investigations showing conflicting results (Li et al., 2011; Li et al., 2016). It is thought these inconsistencies are due to the composition and abundance of gut microbial taxa associated with the parasitic helminths being specific to each species (Kreisinger et al., 2015). A higher species richness may benefit the host, as higher species richness of the gut microbiota has been associated with ‘healthier’ gut homeostasis (Sepehri et al., 2007; Monira et al., 2012; Lin et al., 2013; Lee et al., 2014; Kreisinger et al., 2015). However, this study was designed to simulate a high, close proximity infection of adult rumen fluke, and so the changes in the microbiome seen here may be indicative of a more local effect on the microbiome. It is likely the effects observed may not be seen across the whole rumen. Additionally, EVs derived from rumen fluke may have a greater effect on the bacterial species of the rumen associated with the epithelium and liquid phases, as rumen fluke affix themselves to the rumen epithelium *via* their posterior sucker (McCowan et al., 1978; Michalet-Doreau et al., 2001; Fuertes et al., 2015). EVs ability to regulate the hosts gut microbiota highlights the potential of utilizing EVs in order to promote survival of key bacterial species such as *F. succinogenes* that play a vital role in degradation of plant biomass and so could lead to improved rumen efficiency (Arntzen et al., 2017). A full antimicrobial analysis of all EV proteins characterized would be beneficial as EVs are known to bind to targets in order to become internalized and release their contents and so these may also be influencing the EVs themselves as well as elucidating mechanisms through which EVs released by *C. daubneyi* manipulate the host microbiota leading to conditions favorable for long term establishment.

## Data Availability Statement

The datasets presented in this study can be found in online repositories. The names of the repository/repositories and accession number(s) can be found below: ProteomeXchange Consortium *via* the PRIDE [1] partner repository with the dataset identifier PXD024182.

## Ethics Statement

The animal study was reviewed and approved by Aberystwyth University ethics committee.

## Author Contributions

NA: data collection, analysis, investigation, methodology, and writing—original draft. AL: experimental design, data collection, and analysis. TW: experimental design and formal analysis. SH: experimental design and technical expertise. HP: technical expertise—LC-MSMS. RM and PB—supervision, writing, review, and editing. All authors contributed to the article and approved the submitted version.

## Funding

This work was supported by the Biotechnology and Biological Sciences Research Council through an IBERS PhD Scholarship award and through Innovate UK (Grant Number: 102108).

## Conflict of Interest

The authors declare that the research was conducted in the absence of any commercial or financial relationships that could be construed as a potential conflict of interest.
